# Uptake of Sulfate from Ambient Water by Freshwater Animals

**DOI:** 10.3390/w12051496

**Published:** 2020-05-23

**Authors:** Michael B. Griffith, James M. Lazorchak, Herman Haring

**Affiliations:** 1 U.S. Environmental Protection Agency, Office of Research and Development, National Center for Environmental Assessment, Cincinnati, OH 45268, USA; 2 U.S. Environmental Protection Agency, Office of Research and Development, Center for Environmental Measurement and Modeling, Cincinnati, OH 45268, USA; 3 U.S. Environmental Protection Agency, Office of Research and Development, National Exposure Research Laboratory, Cincinnati, OH 45268, USA; 4 Pegasus Technical Services, Inc., Cincinnati, OH 45268, USA

**Keywords:** sulfate, freshwater, uptake, efflux, fish, invertebrates

## Abstract

To better understand how the sulfate (SO_4_^2−^) anion may contribute to the adverse effects associated with elevated ionic strength or salinity in freshwaters, we measured the uptake and efflux of SO_4_^2−^ in four freshwater species: the fathead minnow (*Pimephales promelas*, Teleostei: Cyprinidae), paper pondshell (*Utterbackia imbecillis*, Bivalvia: Unionidae), red swamp crayfish (*Procambarus clarkii*, Crustacea: Cambaridae), and two-lined mayfly (*Hexagenia bilineata*, Insecta: Ephemeridae). Using *δ(*^*34*^*S*/^*32*^*S)* stable isotope ratios and the concentrations of S and SO_4_^2−^, we measured the SO_4_^2−^ influx rate (*J*_*in*_), net flux (*J*_*net*_), and efflux rate (J_out_) during a 24 h exposure period. For all four species, the means of *J*_*in*_ for SO_4_^2−^ were positive, and *J*_*in*_ was significantly greater than 0 at both target SO_4_^2−^ concentrations in the fish and mollusk and at the lower SO_4_^2−^ concentration in the crayfish. The means of *J*_*out*_ and *J*_*net*_ were much more variable than those for *J*_*in*_, but several species by target SO_4_^2−^ concentration combinations for *J*_*out*_ and *J*_*net*_, were negative, which suggests the net excretion of SO_4_^2−^ by the animals. The results of our experiments suggest a greater regulation of SO_4_^2−^ in freshwater animals than has been previously reported.

## 1. Introduction

Water quality benchmarks were recently developed for elevated total ion concentrations in freshwaters using specific conductance as a measurement endpoint to protect aquatic life [1,2]. However, there is still uncertainty about how different ions contribute to the adverse effects associated with elevated total ionic strength or salinity on freshwater biota, and some studies have suggested a more traditional approach where the concentrations of specific anions should be the targets of chemical monitoring and ambient water quality criteria [3–6]. The predominant form of dissolved sulfur (S) in water is the anion, sulfate (SO_4_^2−^), which can be especially elevated in waters affected by mining. Mining exposes sulfide-rich minerals, such as pyrite associated with coal mining and other metal sulfides associated with hard rock metal mining [7–10]. Under oxic conditions, these sulfides are rapidly transformed into SO_4_^2−^.

Several studies have recently attempted to assess the ecotoxicity of SO_4_^2−^ with standard bioassays that have exclusively used Na_2_SO_4_ [11–14]. The objective of at least some of these studies is the compilation of sufficient bioassay data for a species sensitivity distribution to develop an ambient water criterion for SO_4_^2−^. These studies attribute many of the observed adverse effects to SO_4_^2−^. This assumes that any effects associated with the concurrently elevated concentrations of Na^+^ are minor, even though the uptake of Na^+^ across epithelial membranes is well known [15–17]. Additionally, the uptake of SO_4_^2−^ across external epithelial membranes, such as gills, occurs and has an ionoregulatory or osmoregulatory adverse effect on these animals. However, because the transport physiology of SO_4_^2−^ in freshwater animals is relatively unstudied, the validity of the second assumption is unknown. Other studies have begun to study the interactions between different anion and cation combinations [18–20].

To support decisions relating to the potential for adverse effects associated with individual major ions, Na^+^, K^+^, Ca^2+^, Mg^2+^, Cl^−^, SO_4_^2−^, and HCO_3_^−^, we previously published a literature review on the ion physiology of freshwater species of four animal groups: teleost fish, crustaceans, aquatic insects, and mollusks [21]. While this review found extensive literature on many of these major ions at least for teleost fish, if not always for the freshwater invertebrates, it identified a data gap related to the ionoregulatory physiology of SO_4_^2−^. Limited data appear to suggest that SO_4_^2−^ is relatively impermeant to the gill membranes of fish [22,23], crustaceans [24,25], or unionid mussels [26]. However, SO_4_^2−^-transporters are present in amphibian skin [27], which functions in ion uptake similarly to the gill membranes of fish, crustaceans, and mollusks or to anal papillae, chloride epithelia, or other epithelial surfaces in aquatic insects [21]. However, see the research on SO_4_^2−^ uptake from the water that has been recently published on aquatic insects by Scheibener et al. [28] and Buchwalter et al. [29].

Like other major ions, SO_4_^2−^ is present in the extracellular fluids (i.e., blood or hemolymph) of freshwater animals. Reported SO_4_^2−^ concentrations include 0.76 ± 0.18 mmol/L (n = 6); 2.58 ± 0.06 (n = 30) and 2.60 ± 0.90 mmol/L (n = 7) in two species of *Anodonta* and zebra mussels (*Dreissena polymorpha*), respectively (Mollusca) [30–32]; 0.94 ± 0.26 for the spinycheek crayfish (*Orconectes limosus*) [33]; and 0.89 and 2.14 mmol/L for white sucker (*Catostomus commersoni*) and rainbow trout (*Oncorhynchus mykiss*), respectively [34]. A greater SO_4_^2−^ concentration of 18.7 mmol/L was observed in freshwater Japanese eels (*Anguilla japonica*) [35], but the authors suggest this catadromous species balances greater SO_4_^2−^ with less Cl^−^ in its blood.

Moreover, SO_4_^2−^ is metabolically active. S is a component of the amino acids methionine and cysteine, which produce S-containing proteins, and a component of other biomolecules, such as sulfated glycosaminoglycans, which are generally ubiquitous in metazoans, including mollusks, crustaceans, insects, and fish [36–39]. In most organisms, the initial step in S assimilation is SO_4_^2−^ activation to 3’-phosphoadenosine 5’-phosphosulfate (PAPS) [40–43].

Freshwater animals are generally hyperregulators that maintain greater ion concentrations in their blood or hemolymph than are found in surrounding freshwaters [44]. As the external medium is hypoosmotic or more dilute than body fluids, these species deal with the continuous diffusional loss of salts and the osmosis of water across their permeable membranes. Therefore, water balance is accomplished by the excretion of dilute waste fluids by their renal systems. Salt concentrations are maintained by the function of various ion transporting proteins in epithelial membranes, such as in the gills, gastrointestinal system, or renal system, that allow the active transport of ions against concentration gradients (i.e., the absorption of ions from the surrounding water or food or the reabsorption of ions from waste fluids, such as urine). Furthermore, most freshwater species, unlike saltwater species, limit their drinking of water, thereby limiting the absorption of water through the gastrointestinal system and dilution of the hemolymph. However, increased water concentrations of some ions, such as SO_4_^2−^, change concentration gradients across the epithelial membranes involved in ionoregulation, such as the gills. This may change SO_4_^2−^ influx across these membranes, increase blood or hemolymph SO_4_^2−^ concentrations, and have osmoregulatory or other adverse effects. However, it is also possible that these epithelial membranes could be impermeant to SO_4_^2−^, and none of the above effects may occur. To test whether SO_4_^2−^ can move across epithelial membranes, we conducted laboratory experiments with a fish, a crustacean, a mollusk, and a mayfly. These experiments used protocols similar to traditional toxicity tests in that the organisms were exposed to reconstituted water with elevated SO_4_^2−^ concentrations. However, the concentrations were not expected to have overtly adverse effects, such as mortality. Moreover, the reconstituted water was made, in part, with enriched Na_2_[^34^S]O_4_ to elevate its *δ(*^*34*^*S*/^*32*^*S)*. After exposure, the whole body stable isotope ratios of S were measured to assess whether the ^*34*^*S* associated with animal tissues increased.

This study was designed to fill a data gap by conducting laboratory experiments with species from four freshwater animal groups (crustaceans, fish, unionid mussels, and aquatic insects) to measure SO_4_^2−^ uptake from ambient waters and the effect of ambient SO_4_^2−^ concentrations on SO_4_^2−^ uptake. Significant SO_4_^2−^ uptake from the water into an animal would show that the epithelial membranes are permeant to SO_4_^2−^, likely via an ion-specific transporter. This SO_4_^2−^ uptake would affect internal SO_4_^2−^ concentrations, would play a direct role in ionoregulation by the animal, and could have adverse effects if elevated in freshwaters.

## 2. Materials and Methods

To test whether SO_4_^2−^ uptake is similar or differs among different major taxa groups of freshwater animals, we conducted parallel experiments using a representative species in each of four taxa groups. The ion transport physiology of these species is unlikely to change with the aquatic developmental stage [21]. Therefore, juvenile fish and crayfish, adult unionid mussels, and later instar mayfly nymphs were used in the experiments. The exposures for each of the four species was conducted during a single 24 h period, but the experiments were conducted at different times between September 2017 and April 2019.

### 2.1. Test Animals

Although we considered, originally, using model species (e.g., Cladocera and Chironomidae), often used by toxicity studies, our methods required larger individuals to provide sufficient biomass for stable isotope analysis. We selected the fathead minnow (*Pimephales promelas* Rafinesque, 1820) which is commonly used as a model for a teleost fish. Less conventional test species included a mollusk, the paper pondshell (*Utterbackia imbecillis* (Say, 1829)); a crustacean, the red swamp crayfish (*Procambarus clarkii* (Girard, 1852)); and an aquatic insect, the two-lined mayfly (*Hexagenia bilineata* (Say, 1824)).

We used fathead minnows from laboratory colonies usually cultured and maintained for toxicity testing according to [45,46]. We fed them live *Artemia* nauplii twice a day at a rate of 1 mL per 20 L tank per day-of-age per feeding with a nauplii suspension of 15 mL of brine shrimp cysts (Brine Shrimp Direct, Ogden, UT, USA) incubated for 24 h at 28 °C in aerated Labline water with 25 mL NaCl added.

We purchased red swamp crayfish from Carolina Biological Supply Co., Burlington, NC, USA. The crayfish were placed in a tank with about 175 L of water aerated with air stones. Lengths of polyvinyl chloride pipe cut in half lengthwise were placed in the aquaria to provide cover for the crayfish and reduce aggression. The crayfish were fed thawed adult *Artemia* ad libitum daily.

We obtained the paper pondshells from the Kentucky Department of Fish and Wildlife Resources’ Center for Mollusk Conservation (Frankfort, KY, USA). The mussels were held in aquaria, with 28 individuals per aquarium, where the water was aerated by an air stone. The mollusks were fed 20 mL of FFAY, an internally made mixture of fish flake food (Tetramin®, Tetra, Blacksburg, VA, USA), alfalfa (from capsule; Nature’s Way, Fargo, ND, USA), and yeast (Flieschmann’s, Oakbrook Terrace, IL, USA); and 15 mL of an algal culture of flagellated algae and diatoms (Shellfish Diet 1800®, Reed Mariculture, Campbell, CA, USA) and 15 mL of alfalfa per tank daily.

We purchased the two-lined mayfly from The Reel Thing Live Bait, Green Bay, WI, USA. The mayfly nymphs were held in aquaria, where the water was aerated by an air stone, and were fed ground cereal grain flake food (Cerophyll®, Ward’s Natural Science Establishment, Inc., Rochester, NY, USA).

Before their use in experiments, we held the fish and invertebrates in dechlorinated and hardness-adjusted municipal tap water (mean ionic composition in mmol L^−1^: 1.04 [Na^+^], 0.05 [K^+^], ~0.25–0.55 [Ca^2+^], 0.43 [Mg^2+^], ≤ 0.01 [Cl^−^], and 0.93 [SO_4_^2−^]; pH: 7.2–8.1; total organic carbon (TOC): ~0.08 mg C L^−1^; and hardness: ~150–180 mg L^−1^ as CaCO_3_). All animals, except the two-lined mayflies, were maintained at a constant temperature of 25 ± 1 °C with a 16:8 h light to dark photoperiod. The two-lined mayflies were maintained at a constant temperature of 15 ± 1 °C.

For at least 24 h before the individual experiments, the animals were acclimated to a modified moderately hard reconstituted water (MMHRW) similar to the R-MHRW of Smith et al. [47] generated by the addition of reagent-grade salts to deionized water produced by a Millipore Super-Q Plus water purification system and bubbled with CO_2_ to dissolve the CaCO_3_, particularly using Na_2_SO_4_ (Table 1). During the 24 h acclimation and test phases, the animals were not fed, while the holding water temperatures were maintained.

### 2.2. Test Water

Upon the initiation of the experiment, the animals were exposed to two concentrations of SO_4_^2−^, with the MMHRW with Na_2_SO_4_ added to increase by 2× (i.e., 0.49 mmol L^−1^) or 5 (1.23 mmol L^−1^) the SO_4_^2−^ concentrations to test whether differing water SO_4_^2−^ concentrations influenced uptake (Table 1). This addition also increased Na^+^ concentrations to 1.03 mmol L^−1^ and 2.51 mmol L^−1^, respectively. None of these concentrations are near the concentrations that have been observed to have acute adverse effects on freshwater animals [6,11,12,48]. Moreover, the Na^+^/K^+^ molar ratios were 22.0 and 53.5, respectively, which are within the range where alterations of this ratio have not been observed to have osmoregulatory effects [49,50].

To observe influx rates, test water made with unenriched reagent grade Na_2_SO_4_ was mixed with the same concentration test water made with 90% atom enriched Na_2_[^34^S]O_4_ (Sigma-Aldrich, Miamisburg, OH, USA) at a ratio of 939 parts of reagent grade test water to 61 parts of 90% atom enriched test water. A test water made with only 90% atom enriched Na_2_[^34^S]O_4_ could not be used without dilution to reduce the enrichment of the ^*34*^*S*. Too strong of a signal from ^*34*^*S* when the samples are analyzed in the mass spectrometer can overload the detectors and cause falsely elevated concentration readings in subsequent samples (R. Venkatapathy, personal communication).

### 2.3. Sampling Methods

#### 2.3.1. Measurements of Animals and Water

Measurements were made on organisms placed in containers with aeration, containing 200 mL, 12 L, 7 L, and 150 mL of the waters for the minnow, unionid mollusk, crayfish, and mayfly, respectively. The volumes were chosen based on the mass loading limits for the animals in static tests [45]. The two-lined mayflies were supplied with short lengths (~38 mm) of 9.5 mm inner-diameter vinyl tubing as artificial burrows [51]. In all four species, the mass of the animal (i.e., ≥10 mg dry mass) was sufficient for chemical analysis, permitting us to measure each individual.

Subsamples of the test waters were taken to measure the SO_4_^2−^ concentrations and *δ(*^*34*^*S*/^*32*^*S)* for each exposure at 0 h, and a water sample was taken from each exposure container to measure the SO_4_^2−^ concentrations after the 24 h exposures. At least 10 extra individuals of each species were sacrificed at 0 h to measure initial whole-body S concentrations and *δ*(^34^S/^32^S). Measurements from 25 separate replicates of each SO_4_^2−^ concentration were collected for each test species. From every exposure, replicate whole-body samples of each test species were taken at the end of the exposure to the enriched stable isotope water and used to measure whole-body S concentrations and *δ(*^*34*^*S*/^*32*^*S).* However, because there was some mortality in the two-lined mayfly experiment, the numbers of replicates were 17 and 22 for the 0.49 and 1.25 mmol L^−1^ SO_4_^2−^ exposures, respectively. Moreover, an error in the initial water SO_4_^2−^ analysis in the mayfly experiment reduced the number of valid measurements of the initial 0.49 mmol L^−1^ water SO_4_^2−^ concentration to 1, precluding valid means of *J*_*out*_ and *J*_*net*_ for the 0.49 mmol L^−1^ SO_4_^2−^ exposure.

At the end of each exposure, the fish, crayfish, or mayfly nymph individuals were removed from the containers, rinsed in deionized water to remove any stable isotope label from the surface, and blotted dry on filter paper. For the unionid mussel, the soft tissue was cut from the shell. The fish, mussels (soft tissue only), and mayfly nymphs were dried overnight at 90 °C, whereas the crayfish were freeze-dried for 14 days to weaken their exoskeletons. The animals were then weighed, homogenized, and powdered to 100–200 μm with a mortar and pestle, stored in vials, and analyzed for total S and *δ(*^*34*^*S*/^*32*^*S)* [52].

#### 2.3.2. Chemical Analysis of Test Water and Animals

The total concentrations of SO_4_^2−^ in the test waters were measured with ion chromatography (EPA Method 300.0) [53]. For the stable isotope analysis of SO_4_^2−^ in the initial test waters, we precipitated the dissolved SO_4_^2−^ in subsamples of the water placed in the test chambers as barium sulfate (BaSO_4_) following Révész et al. [54].

The total S concentration of the dried animal tissues was measured using an elemental analyzer. The molar ratios of ^34^S/^32^S, reported as the deviation of this ratio from the international standard, Canon Diablo Troilite, or *δ(*^*34*^*S*/^*32*^*S)* [55], were measured in both the BaSO_4_ precipitates and dried animal tissues with an elemental analyzer connected to a continuous-flow 20–20 gas source stable isotope ratio mass spectrometer [52,54]. For ^*34*^*S* analyses, the sample was placed in a tin capsule with vanadium dioxide for combustion, the combustion reactor was held at 1080 °C, and the reaction tube contained tungsten oxide on alumina as an oxidative catalyst and copper metal to remove excess O_2_ subsequent to combustion. The S in the sample was converted to SO_2_ and, along with N_2_ and CO_2_, was passed through two H_2_O traps. The purified gases were then separated in a 30 cm, 0.5” OD, QS GC column held at 45 °C and passed into the mass spectrometer—N_2_ and CO_2_ first, and then SO_2_. Approximately one-third of the SO_2_ was cracked to SO in the source, allowing ^*34*^*S* to be measured by continuously monitoring masses 48, 49, and 50. The mass peaks were plotted, the area under each mass peak was determined, and the isotope ratios were calculated. These ratios were referenced to ratios determined on in-house reference materials analyzed in the same analytical run. The raw ratio data were corrected for drift over the course of the run along with blank/linearity effects, and if present, then normalized to the international standards. Analytical precisions, based on the replicate analyses of international reference materials, were ±0.3‰ for δ^34^S.

#### 2.3.3. Compartmental Analysis to Calculate the Influx and Efflux of Sulfate

The SO_4_^2−^, total S, and δ^34^S ratio data were used in a compartmental analysis of a single pool system for using stable isotope tracer data [56,57] modified from models using radioisotope data for ion uptake by fish and crayfish [58–60]. Compartmental analysis models the movement of a solute between two compartments based on diffusion and mass conservation. In this system, we are measuring the movement of SO_4_^2−^ between the surrounding water and the animal across semipermeable epithelial cell membranes that may be facilitated by the presence of SO_4_^2−^-specific transporter proteins. This is because the SO_4_^2−^ in the test water can be traced with the artificially elevated levels of ^*34*^*S* by the SO_4_^2−^-influx rate (J_in_). However, because only the change in the SO_4_^2−^ concentration in the test waters was used to measure the net SO_4_^2−^-flux rate (J_net_), the SO_4_^2−^-efflux rate (J_out_) may include S from sources other than the test water, such as food.

#### 2.3.4. Calculations

*J*_*net*_ was measured as the change in the water SO_4_^2−^ concentration during the exposure period (*t* = 1 day):

(1) $$J_{net}=\frac{{\left[SO_{4}^{2-}\right]}_{0}\times V_{0}-{\left[SO_{4}^{2-}\right]}_{t}\times V_{t}}{M\times t}$$

where [*SO*_4_^2−^]_0_ and [*SO*_4_^2−^]_*t*_ are the concentrations of SO_4_^2−^ in the water (μmol L^−1^) at the beginning and end of the exposure period, respectively; *V* is the volume of the water in the chamber (L) measured at *t* = 0 and *t* ≈ 24 h to account for evaporation; *M* is the mass (g) of the animal placed into the container; and *t* is the length of the exposure period (days).

*J*_*in*_ was measured as the changes in the fractional molar abundance of ^*34*^*S* and total S concentration in the animal during the exposure interval relative to the initial fractional molar abundance of ^*34*^*S* in the test waters:

(2) $$J_{in}=\frac{\left[X{\left({}^{34}S\right)}_{int\left(t\right)}\times {\left[S\right]}_{int\left(t\right)}\right]-\left[X{\left({}^{34}S\right)}_{int\left(0\right)}\times {\left[S\right]}_{int\left(0\right)}\right]}{\left[X{\left({}^{34}S\right)}_{\text{bath}}\times t\right]}$$

where *X*(^34^*S*)_*int*(0)_ is the initial fractional molar abundance of ^*34*^*S* in the animal, [*S*]_*int*(0)_ is the initial concentration of S in the animal (μmol g^−1^), *X*(^34^*S*)_*int*(*t*)_ is the fractional molar abundance of ^*34*^*S* at the end of the exposure, [*S*]_*int*(*t*)_ is the concentration of S in the animal at the end of the exposure (μmol/g^−1^), *X*(^34^*S*)_*bath*_ is the fractional molar abundance of ^*34*^*S* in the test waters, and *t* is the length of the exposure period (day).

The fractional molar abundance of ^*34*^*S* or *X*(^34^*S*) for a sample is calculated from the molar ratio of ^*34*^*S* to ^*32*^*S* or *R(*^*34*^*S*/^*32*^*S)* for the sample by:

(3) $$X\left({}^{34}S\right)=\frac{R\left({}^{34}S{/}^{32}S\right)}{1+R\left({}^{34}S{/}^{32}S\right)}$$

and *R(*^*34*^*S*/^*32*^*S)* is calculated from the reported *δ(*^*34*^*S*/^*32*^*S)* by:

(4) $$R\left({}^{34}S{/}^{32}S\right)=\frac{\delta \left({}^{34}S{/}^{32}S\right)\times N{\left({}^{34}S\right)}_{std}/N{\left({}^{32}S\right)}_{std}}{1000}+N{\left({}^{34}S\right)}_{std}/N{\left({}^{32}S\right)}_{std}$$

where *N(*^*34*^*S)*_*std*_/*N(*^*32*^*S)*_*std*_ is the molar ratio of the heavy stable isotope in the standard material, Canyon Diablo Troilite, which by convention is assigned a *N(*^*34*^*S)*/*N(*^*32*^*S)* of 0.045005 [61]. The values are divided by 1000, because *δ(*^*34*^*S*/^*32*^*S)* is reported in parts per mille relative to the standard material.

*J*_*out*_ is calculated as the difference between *J*_*net*_ and *J*_*in*_ [58]:

(5) $$J_{out}=J_{net}-J_{in}$$

#### 2.3.5. Statistical Analysis

As the measurements of SO_4_^2−^ and *δ(*^*34*^*S*/^*32*^*S)* in the water and in animals at the beginning of the exposures were made on and summarized for replicate subsamples, the variation associated with these measurements was pooled as appropriate with the variation of the calculated variables, *J*_*in*_, *J*_*out*_, and *J*_*net*_. Then, the calculated variables for each species were tested to determine whether each variable was significantly different from 0 using a t-test (PROC TTEST, SAS Institute, Cary, NC, USA). Because six variable-by-concentration combinations were tested for each species, a Bonferroni adjustment of p = 0.0083 was used.

## 3. Results

The measured SO_4_^2−^ concentrations in the artificial water used with the different species were variable compared to the target concentrations (Table 2). The measured *δ(*^*34*^*S*/^*32*^*S)* for the artificial waters (Table 2) were more than 100 times the initial measured *δ(*^*34*^*S*/^*32*^*S)* of the animal tissues, which were +11.386 ± 0.090, −1.420 ± 0.910, −2.304 ± 0.987, and −4.617 ± 0.530 for the fathead minnows, paper pondshells, red swamp crayfish, and two-lined mayflies, respectively. The summary statistics for all the variables used in the compartmental analyses may be found in Table S1.

**Figure 1.**
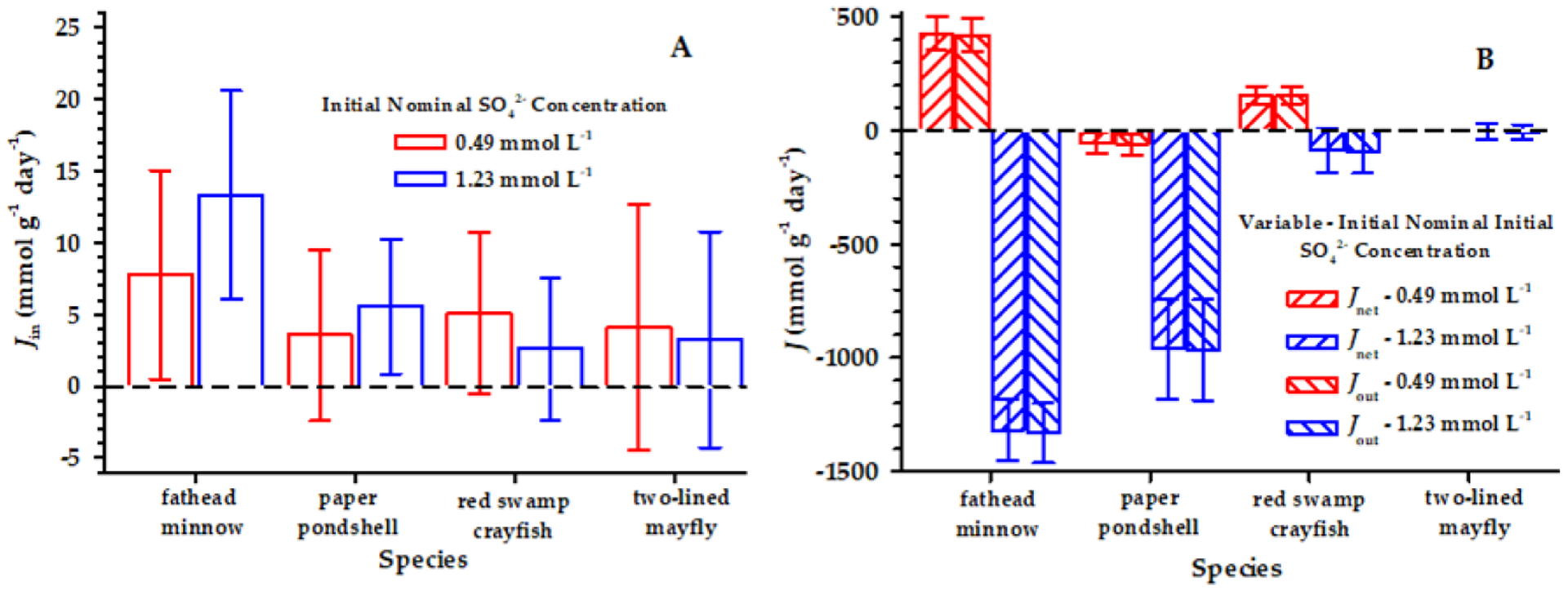
Mean SO_4_^2−^ influx (J_in_) **(A)**, efflux (J_out_), and net flux (J_net_) **(B)** (μmol g^−1^ day^−1^) for each animal species—the fathead minnow, paper pondshell, red swamp crayfish, and two-lined mayfly—at the two target SO_4_^2−^ concentrations of 0.49 mmol L^−1^ and 175 mmol L^−1^. The error bars represent ±1 standard error. Because of some mortality occurring in the mayfly experiment, the numbers of replicates were 17 and 22 for the 0.49- and 1.25-mmol L^−1^ SO_4_^2−^ exposures, respectively. Moreover, an error in the initial water SO_4_^2−^ analysis in the mayfly experiment reduced the number of valid measurements for the initial 0.49 mmol L^−1^ water SO_4_^2−^-concentration to 1, precluding a valid mean of *J*_*out*_ and *J*_*net*_ for the 0.49 mmol L^−1^ SO_4_^2−^ exposure.

The means of *J*_*in*_ for SO_4_^2−^ were positive for all the species and ranged from 2.14 to13.32 μmol g^−1^ day^−1^ among the species and two nominal sulfate concentrations (Figure 1). The *J*_*in*_ in 5 of 50, 12 of 50, 13 of 50, and 8 of 39 individual exposures of the fish, mollusk, crayfish, and mayfly were negative, and there were large confidence bounds around the means (i.e., the coefficient of variation ranges from 0.54 to 3.63). In part, notable variance was added to the means of *J*_*in*_ because of unexpected variation in the measurements of the initial animal S concentrations (Table 3), but *J*_*in*_ was significantly greater than 0 at both target SO_4_^2−^ concentrations in the fish and mollusk and at the lower SO_4_^2−^ concentration (i.e., 0.49 mmol L^−1^) in the crayfish (Table 4). Additionally, J_in_ increased between the two SO_4_^2−^ concentrations in the fish and mollusk (Figure 1), although the increase was statistically significant only for the fish (df = 48, *t* = 2.70, p = 0.009) and the mollusk (df = 48, *t* = 1.13, p = 0.20).

The means of *J*_*out*_ and *J*_*net*_ were much more variable but suggested a net excretion of SO_4_^2−^ by the four species (Figure 1, Table 4). Part of this variation was the result of unexpected variation in the measurements of the initial water concentrations of SO_4_^2−^ (Table 3).

## 4. Discussion

Because we measured an increase in whole animal *R(*^*34*^*S*/^*32*^*S)* in animals exposed to water where the added SO_4_^2−^ was highly enriched with ^*34*^*S*, *J*_*in*_ measured the uptake of SO_4_^2−^ from the water. Presumably, the SO_4_^2−^ moved through SO_4_^2−^-transporters on external epithelial membranes, such as the gills, chloride cells, or integument, because other ions commonly move through epithelial membranes via various intercellular pathways involving various ion transporters. Although our methods used whole animal assays and therefore did not definitively distinguish between external surfaces and internal tissues, the measured *J*_*in*_, when expressed in the same units, are of a similar range of magnitude as the measurements of the uptake of other ions, including Cl^−^ anions, at similar water concentrations [62–65]. Even though our methods do not distinguish between ionocyte-mediated transport and paracellular transport, which has been described for other ions, paracellular transport does not generally occur against concentration gradients, and we expect that it would take longer than 24 h for the equilibration of the isotope between the test solution and the internal milieu without the aid of facilitated or active transport [66–69].

The larger *J*_*out*_ suggests that there is an internal pool of SO_4_^2−^ supplied by sources in addition to uptake from the water, such as from food [70,71]. By measuring the change in the SO_4_^2−^ concentration in the water, *J*_*out*_ and *J*_*net*_ measure all the effluxes of SO_4_^2−^ between the animals and the water, including renal excretion.

A number of other studies have observed SO_4_^2−^ transporters in aquatic animals, primarily associated with internal epithelial membranes. In teleost fish, two types of epithelial SO_4_^2−^ transporters, a Na^+^/SO_4_^2−^-cotransporter (NaS1, SLC13 family) and a SO_4_^2−^/anion-exchanger (Sat1, SLC26 family), have been identified in the proximal tubules of the kidneys that are primarily involved in SO_4_^2−^ excretion and resorption [35,72,73]. The functional analysis of freshwater mollusks suggests a similar renal regulation of SO_4_^2−^ [26,30]. In mosquito (*Aedes campestris*) larvae from saline lakes, transporters associated with the Malpighian tubules excrete SO_4_^2−^ [74,75]. In marine Atlantic lobsters (*Homarus americanus*), SO_4_^2−^ transporters associated with hepatopancreatic epithelia excrete SO_4_^2−^ in exchange for either C_2_H^2−^ or Cl^−^ [76–79]. The Na^+^/SO_4_^2−^-cotransporter, NaS1, was also detected in the intestinal tissue of zebrafish (*Danio rerio*) but not gill tissues [72]. However, our experiments were not designed to identify the types of SO_4_^2−^ transporters beyond their role in uptake from the ambient water.

The amino acid cysteine and S-containing proteins are synthesized in metazoans from the amino acid methionine. While the initial step is SO_4_^2−^ activation to PAPS, S is added to methionine by the sulfate assimilatory reduction of PAPS, a pathway not found in metazoans [40–43]. Therefore, these amino acids are sources of S in animals via ingestion. In other biomolecules, sulfate groups are transferred from PAPS by a sulfonation pathway, which is found in metazoans [41,43]. Therefore, inorganic SO_4_^2−^ from some source is required by animals. The renal reabsorption of SO_4_^2−^ is likely part of the source, but the source is also supplied by the direct uptake of SO_4_^2−^ from the water, which we observed in our experiments.

In freshwater nymphs of Plecoptera, Ephemeroptera, and Trichoptera, Scheibener et al. [28] measured the uptake of SO_4_^2−^ and found that this uptake was inhibited by increased Na^+^ water concentrations, suggesting the presence of a Na^+^/SO_4_^2−^-cotransporter. Buchwalter et al. [29] identified similar SO_4_^2−^ transporters in the mayfly, *Neocloeon trangulifer*, but the localization of these transporters was not determined. This research also observed that SO_4_^2−^ uptake increased with an increasing SO_4_^2−^ water concentration.

In conclusion, our study, along with studies from the Buchwalter laboratory [28,29], suggests that there is direct uptake of SO_4_^2−^ from the water in these four groups of freshwater animals. Additionally, there is some evidence that this uptake may increase with the water concentration of SO_4_^2−^. However, the uptake of SO_4_^2−^ from the water is not the only source of S, and S from food likely contributes to the SO_4_^2−^ excreted by these animals. Therefore, elevated water SO_4_^2−^ may interact with other ions to have ionoregulatory effects in freshwater animals that could cause the effects observed by more traditional ecotoxicological studies.

A next step for research on any of these freshwater animals would be to sequence, locate, and functionally characterize any SO_4_^2−^-transporters on their gills or other external epithelial membranes, as has been done for other ions [80–83]. Such information would clarify the potential interactions between SO_4_^2−^ and other ions, such as Na^+^, when these ions are elevated in freshwaters. This will more completely identify the potential pathways for adverse outcomes [84] for elevated SO_4_^2−^ in freshwaters and better support risk assessments, leading to the development of water-quality benchmarks or criteria.

## Supplementary Material

Supplement1

## Figures and Tables

**Table 1.** Concentrations of salts in the modified moderately hard reconstituted water (MMHRW) used in the experiment along with the resulting target mmol L^−1^ of the major ions, along with hardness and the molar [Na^+^]/[K^+^] ratios. Only the concentrations of Na^+^ and SO_4_^2−^ were increased in the MMHRW.

Water	Salt	mg L^−1^	Ions	mmol L^−1^
Acclimation	CaCO_3_	71.0	Na^+^	0.54
	NaHCO_3_	4.0	K^+^	0.05
	MgCl_2_·6H_2_0	59.4	Ca^2+^	0.71
	KCl	3.5	Mg^2+^	0.29
	Na_2_SO_4_	35.0	HCO_3_^−^	1.47
			Cl^−^	0.34
			SO_4_^2−^	0.25
			Hardness (mg/L, CaCO_4_)	100.2
			Molar [Na^+^]/[K^+^]	11.5
0.49 mmol L^−1^ SO_4_^2−^	Na_2_SO_4_	70.0	Na^+^	1.03
			SO_4_^2−^	0.49
			Hardness (mg/L, CaCO_4_)	100.2
			Molar [Na^+^]/[K^+^]	22.0
1.23 mmol L^−1^ SO_4_^2−^	Na_2_SO_4_	175.0	Na^+^	2.51
			SO_4_^2−^	1.23
			Hardness (mg L^−1^, CaCO_4_)	100.2
			[Na^+^]/[K^+^]	53.5

MMHRW = modified moderately hard reconstituted water.

**Table 2.** Measured initial characteristics of SO_4_^2−^ in the test water for each species exposure. The column headings are the nominal concentrations of SO_4_^2−^. The values are the sample mean ± 1 standard error. [SO_4_^2−^] (mmol L^−1^) is the concentration of SO_4_^2−^, and *δ(*^*34*^*S*/^*32*^*S)* (‰) is molar ratio of ^*34*^*S*/^*32*^*S* of the SO_4_^2−^.

Variable	Species	0.49 mmol L^−1^		1.23 mmol L^−1^	
		n	Value	n	Value
[SO_4_^2−^] (mmol L^−1^)	Fathead minnow	5	0.52 ± 0.03	5	1.67 ± 0.03
	Paper pondshell	5	0.35 ± 0.01	5	0.98 ± 0.06
	Red swamp crayfish	10	0.45 ± 0.01	10	1.15 ± 0.01
	Two-lined mayfly	1	0.48	3	1.19 ± 0.04
*δ(*^*34*^*S*/^*32*^*S)* (‰)	Fathead minnow	5	+1377.6 ± 1.3	5	+1433.9 ± 0.8
	Paper pondshell	5	+1432.7 ± 34.1	5	+1452.6 ± 0.5
	Red swamp crayfish	5	+1475.6 ± 2.8	5	+1461.3 ± 1.0
	Two-lined mayfly	5	+1411.6 ± 3.0	5	+1365.7 ± 4.4

**Table 3.** Proportion of variance in the J value means contributed by the variances of the mean values for component variables. The proportions of variance for other component variables were <0.001, or means were not used in the calculations. [S]_*int*(0)_ is the mean calculated initial S concentration (μmol g^−1^) of each animal, and [SO_4_^2−^]_0_ is the measured concentrations of SO_4_^2−^ in the water (μmol L^−1^) at the beginning of the exposure. *J*_*in*_, *J*_*out*_, and *J*_*net*_ are the influx rates, efflux rates, and net flux of SO_4_^2−^(μmol g^−1^ day^−1^), respectively, for each species.

Species	Target SO_4_^2−^ Concentration	*J* value	Variable	Proportion of Variance
Fathead minnow	0.49 mmol L^−1^	*J*_*in*_	[*S*]_*int*(0)_	0.603
		*J*_*out*_	${\left[SO_{4}^{2-}\right]}_{0}$	0.089
			[*S*]_*int*(0)_	0.015
		*J*_*net*_	${\left[SO_{4}^{2-}\right]}_{0}$	0.090
	1.23 mmol L^−1^	*J*_*in*_	[*S*]_*int*(0)_	0.584
		*J*_*out*_	${\left[SO_{4}^{2-}\right]}_{0}$	0.583
			[*S*]_*int*(0)_	0.006
		*J*_*net*_	${\left[SO_{4}^{2-}\right]}_{0}$	0.058
Paper pond shell	0.49 mmol L^−1^	*J*_*in*_	[*S*]_*int*(0)_	0.172
		*J*_*out*_	${\left[SO_{4}^{2-}\right]}_{0}$	0.046
			[*S*]_*int*(0)_	0.021
		*J*_*net*_	${\left[SO_{4}^{2-}\right]}_{0}$	0.047
	1.23 mmol L^−1^	*J*_*in*_	[*S*]_*int*(0)_	0.519
		*J*_*out*_	${\left[SO_{4}^{2-}\right]}_{0}$	0.059
			[*S*]_*int*(0)_	0.001
		*J*_*net*_	${\left[SO_{4}^{2-}\right]}_{0}$	0.059
Red swamp crayfish	0.49 mmol L^−1^	*J*_*in*_	[*S*]_*int*(0)_	0.582
			${\left[SO_{4}^{2-}\right]}_{0}$	0.028
		*J*_*out*_	[*S*]_*int*(0)_	0.012
		*J*_*net*_	${\left[SO_{4}^{2-}\right]}_{0}$	0.072
	1.23 mmol L^−1^	*J*_*in*_	[*S*]_*int*(0)_	0.747
			${\left[SO_{4}^{2-}\right]}_{0}$	0.002
		*J*_*out*_	[*S*]_*int*(0)_	0.002
		*J*_*net*_	${\left[SO_{4}^{2-}\right]}_{0}$	0.005
Two-lined mayfly	0.49 mmol L^−1^	*J*_*in*_	[*S*]_*int*(0)_	0.890
	1.23 mmol L^−1^	*J*_*in*_	[*S*]_*int*(0)_	0.941
		*J*_*out*_	${\left[SO_{4}^{2-}\right]}_{0}$	0.346
			[*S*]_*int*(0)_	0.084
		*J*_*net*_	${\left[SO_{4}^{2-}\right]}_{0}$	0.380

**Table 4.** T-value (df and p) for the test of the H_0_ that each mean *J* = 0. m*J*_*in*_, m*J*_*out*_, and m*J*_*net*_ are the mean influx rate, mean efflux rate, and mean net flux of SO_4_^2−^, respectively, for each species. Because of the multiple tests for each animal species, the Bonferroni adjustment of *p* is 0.0083. The *t*-values that indicate a mean J value statistically different from 0 are in bold.

Species	Target SO_4_^2−^ Concentration	H_0_: m*J*_*in*_ = 0 *t*-Value (df, p)	H_0_: m*J*_*out*_ = 0 *t*-Value (df, p)	H_0_: m*J*_*net*_ = 0 *t*-Value (df, p)
Fathead minnow	0.49 mmol/L	**5.32 (24, <0.0001)**	**27.88 (24, <0.0001)**	**28.60 (24, <0.0001)**
	1.23 mmol/L	**9.15 (24, <0.0001)**	**−49.38 (23, <0.0001)**	**−48.51 (23, <0.0001)**
Paper pondshell	0.49 mmol/L	**2.98 (24, 0.0066)**	**−6.38 (24, <0.0001)**	**−6.04 (24, <0.0001)**
	1.23 mmol/L	**5.91 (24, <0.0001)**	**−21.78 (24, <0.0001)**	**−21.74 (24, <0.0001)**
Red swamp crayfish	0.49 mmol/L	**4.50 (24, 0.0001)**	**19.26 (24, <0.0001)**	**20.34 (24, <0.0001)**
	1.23 mmol/L	2.60 (24, 0.0155)	**−4.80 (24, <0.0001)**	**−4.68 (24, <0.0001)**
Two-lined mayfly	0.49 mmol/L	1.95 (17, 0.0680)		
	1.23 mmol/L	1.97 (21, 0.0623)	−0.33 (2, 0.77)	−0.16 (2, 0.89)
