# Supplementary material for: Uptake of Sulfate from Ambient Water by Freshwater Animals
Source: Water (Basel). Author manuscript; Available in PMC 2021 May 23. (PMC7376752; doi:10.3390/w12051496)
Supplement: Supplement1 [file NIHMS1603509-supplement-Supplement1.pdf]

Table S1. Summary statistics (mean, standard error (SE), and n for all variables used in the equations to estimate J_{in}, J_{out}, and J_{net} (µmol g^{-1} day^{-1}). In cases where n was normally ≤ 10, the mean value was used in the equations, but if n was greater, the individual values were used in the equations, and a mean was calculated for the result.

Species	Nominal concentration	$[SO_4^{2-}]_0$ (µmol L^{-1})			V_0 (L)			M (g)		
		mean	SE	n	mean	SE	n	mean	SE	n
Pimephales promelas	0.49 mmol L^{-1} SO_4^{2-}	518.537	26.255	5	0.19678	0.00032	25	0.01151	0.00060	25
Pimephales promelas	1.25 mmol L^{-1} SO_4^{2-}	1673.758	32.738	5	0.19761	0.00012	25	0.01150	0.00069	25
Utterbackia imbecillis	0.49 mmol L^{-1} SO_4^{2-}	358.733	10.163	5	6	0	25	1.01784	0.04558	25
Utterbackia imbecillis	1.25 mmol L^{-1} SO_4^{2-}	975.400	55.162	5	6	0	25	0.90156	0.04314	25
Procambarus clarkii	0.49 mmol L^{-1} SO_4^{2-}	453.813	10.411	10	6.96884	0.00085	25	1.28851	0.07104	25
Procambarus clarkii	1.25 mmol L^{-1} SO_4^{2-}	1150.344	6.964	10	6.99352	0.02519	25	1.32250	0.07028	25
Hexagenia bilineata	0.49 mmol L^{-1} SO_4^{2-}	477.350	NA	1	0.19768	0.00018	17	0.04856	0.00330	17
Hexagenia bilineata	1.25 mmol L^{-1} SO_4^{2-}	1193.375	40.188	3	0.19699	0.00015	22	0.05477	0.0025404	22

Species	Nominal concentration	$[SO_4^{2-}]_t$ (µmol L^{-1})			V_t (L)			t (day)		
		mean	SE	n	mean	SE	n	mean	SE	n
Pimephales promelas	0.49 mmol L^{-1} SO_4^{2-}	517.049	3.320	25	0.18856	0.00150	25	1.0068	0.0017	25
Pimephales promelas	1.25 mmol L^{-1} SO_4^{2-}	1793.788	8.437	24	0.19187	0.00073	25	1.0185	0.0005	25
Utterbackia imbecillis	0.49 mmol L^{-1} SO_4^{2-}	373.340	7.215	25	5.91744	0.00396	25	1.0261	0.0027	25
Utterbackia imbecillis	1.25 mmol L^{-1} SO_4^{2-}	1109.308	25.977	25	5.91944	0.00239	25	1.0203	0.0019	25
Procambarus clarkii	0.49 mmol L^{-1} SO_4^{2-}	440.110	6.383	25	6.78184	0.01591	25	1.0219	0.0024	25
Procambarus clarkii	1.25 mmol L^{-1} SO_4^{2-}	1197.717	18.429	25	6.78872	0.01783	25	1.0386	0.0030	25
Hexagenia bilineata	0.49 mmol L^{-1} SO_4^{2-}	441.770	7.253	17	0.19660	0.00073	17	1.0062	0.0005	17
Hexagenia bilineata	1.25 mmol L^{-1} SO_4^{2-}	1195.774	5.957	22	0.19622	0.00022	22	1.0145	0.0012	22

Table S1 (continued). Summary statistics (mean, standard error (SE), and n for all variables used in the equations to estimate J_{in}, J_{out}, and J_{net} ($\mu mol\ g^{-1}\ day^{-1}$). In cases where n was normally ≤ 10, the mean value was used in the equations, but if n was greater, the individual values were used in the equations, and a mean was calculated for the result.

Species	Nominal concentration	$X(^{34}S)_{int(t)}$			$[S]_{int(t)}$ ($\mu mol\ g^{-1}$)			$\delta(^{34}S/^{32}S)_{int(t)}$ (‰)		
		mean	SE	n	mean	SE	n	mean	SE	n
Pimephales promelas	0.49 mmol L^{-1} SO_4^{2-}	0.043797	0.000018	25	257.893	5.152	25	17.7230	0.4471	25
Pimephales promelas	1.25 mmol L^{-1} SO_4^{2-}	0.044070	0.000040	25	269.567	4.860	25	24.3733	0.9845	25
Utterbackia imbecillis	0.49 mmol L^{-1} SO_4^{2-}	0.043865	0.000084	25	219.448	5.704	25	19.3853	2.0394	25
Utterbackia imbecillis	1.25 mmol L^{-1} SO_4^{2-}	0.043680	0.000068	25	225.128	4.914	25	14.8856	1.6491	25
Procambarus clarkii	0.49 mmol L^{-1} SO_4^{2-}	0.042956	0.000046	25	142.482	5.097	25	-2.6814	1.1106	25
Procambarus clarkii	1.25 mmol L^{-1} SO_4^{2-}	0.043193	0.000040	25	135.960	4.339	25	3.0712	0.9627	25
Hexagenia bilineata	0.49 mmol L^{-1} SO_4^{2-}	0.043002	0.000021	19	267.577	4.332	19	-1.5793	0.5095	19
Hexagenia bilineata	1.25 mmol L^{-1} SO_4^{2-}	0.043185	0.000040	22	263.914	3.927	22	2.8788	1.2090	22

Species	Nominal concentration	$X(^{34}S)_{bath}$			$\delta(^{34}S/^{32}S)_{bath}$ (‰)		
		mean	SE	n	mean	SE	n
Pimephales promelas	0.49 mmol L^{-1} SO_4^{2-}	0.096660	0.000053	5	1377.59	1.44	5
Pimephales promelas	1.25 mmol L^{-1} SO_4^{2-}	0.098725	0.000028	5	1433.94	0.77	5
Utterbackia imbecillis	0.49 mmol L^{-1} SO_4^{2-}	0.098672	0.001243	5	1432.67	34.14	5
Utterbackia imbecillis	1.25 mmol L^{-1} SO_4^{2-}	0.099408	0.000017	5	1452.63	0.47	5
Procambarus clarkii	0.49 mmol L^{-1} SO_4^{2-}	0.100245	0.000109	5	1475.59	2.98	5
Procambarus clarkii	1.25 mmol L^{-1} SO_4^{2-}	0.099724	0.000044	4	1461.29	1.22	4
Hexagenia bilineata	0.49 mmol L^{-1} SO_4^{2-}	0.097907	0.000082	5	1411.58	2.23	5
Hexagenia bilineata	1.25 mmol L^{-1} SO_4^{2-}	0.096225	0.000137	5	1365.74	3.72	5

Table S1 (continued). Summary statistics (mean, standard error (SE), and n for all variables used in the equations to estimate J_{in}, J_{out}, and J_{net} (µmol g^{-1} day^{-1}). In cases where n was normally ≤ 10, the mean value was used in the equations, but if n was greater, the individual values were used in the equations, and a mean was calculated for the result.

Species	Nominal Concentration	$X(^{34}S)_{int(0)}$			$[S]_{int(0)}$ (µmol g^{-1})			$\delta(^{34}S/^{32}S)_{int(0)}$ (‰)		
		mean	SE	n	mean	SE	n	mean	SE	n
Pimephales promelas	both	0.043536	0.000004	10	242.165	10.236	10	11.3863	0.0899	10
Utterbackia imbecillis	both	0.043008	0.000038	10	215.481	6.115	10	-1.4201	0.9103	10
Procambarus clarkii	both	0.042972	0.000041	20	130.423	4.801	20	-2.3040	0.9869	20
Hexagenia bilineata	both	0.042876	0.000065	10	258.431	10.668	10	-4.6170	0.5008	10
